# Development of Nested PCR and Duplex Real-Time Fluorescence Quantitative PCR Assay for the Simultaneous Detection of *Theileria equi* and *Babesia caballi*

**DOI:** 10.3389/fvets.2022.873190

**Published:** 2022-05-18

**Authors:** Kunying Lv, Yiwei Zhang, Yixin Yang, Zheng Liu, Liang Deng

**Affiliations:** ^1^Department of Animal Genetics, Breeding and Reproduction, College of Animal Science and Veterinary Medicine, Shenyang Agricultural University, Shenyang, China; ^2^Key Laboratory of Livestock Infectious Diseases in Northeast China, Ministry of Education, Key Laboratory of Zoonosis, College of Animal Science and Veterinary Medicine, Shenyang Agricultural University, Shenyang, China

**Keywords:** equine piroplasmosis, *Theileria equi*, *Babesia caballi*, nested PCR, duplex real-time fluorescence quantitative PCR, simultaneous detection

## Abstract

Equine piroplasmosis (EP) is a type of blood protozoan disease caused by tick-borne parasites, *Theileria equi* (*T. equi*), *Babesia caballi* (*B. caballi*) and *Theileria haneyi*. While many studies have been conducted on EP diagnosis, diagnostic methods exhibiting high sensitivity and specificity remain lacking. Therefore, nested PCR (nPCR) and duplex real-time fluorescence quantitative PCR (qPCR) that can simultaneously detect both *T. equi* and *B. caballi* causing agents were established and compared. The two techniques were used to analyze 36 horse blood samples for EP. This set of samples was also detected by a multinested PCR (mnPCR) targeting the *EMA-1* gene of *T. equi* and the *RAP-1* gene of *B. caballi*. By nPCR, duplex real-time fluorescence qPCR and mnPCR, infections with *B. caballi* were detected in 16.67% (6/36), 2.78% (1/36), 19.44% (7/36) of the horses, respectively. The *T. equi* prevalence was 58.33% (21/36) by the nPCR, 33.33% (12/36) by the duplex real-time fluorescence qPCR and 2.78% (1/36) by the mnPCR. The overall prevalence of infection with mixed parasites by nPCR was 5.56% (2/36), by duplex real-time fluorescence qPCR was 2.78% (1/36) and by mnPCR 0% (0/36). Results suggest that nPCR can detect *T. equi* and *B. caballi* positive samples with good specificity and sensitivity, although distinguishing between the two parasites requires an electrophoresis with 4% agarose gels. The duplex real-time fluorescence qPCR can readily distinguish between *T. equi* and *B. caballi* infection, but with low sensitivity.

## Graphical Abstract

Schematic description of nested PCR and duplex real-time fluorescence quantitative PCR assay for simultaneous detection of *B. caballi* and *T. equi* in horse blood specimens. 
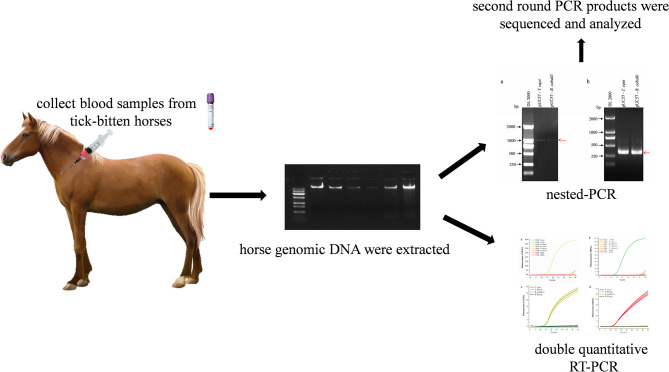

## Introduction

Equine piroplasmosis (EP) is a type of blood protozoan disease caused by the haemoprotozoan parasites, *Theileria equi* (*T. equi*), *Babesia caballi* (*B. caballi*) (1), and the newly identified species *Theileria haneyi* (2). EP can be characterized by fever, hemolytic anemia, hemoglobinuria, icterus, splenomegaly and occasionally death, resulting in significant economic loss to the equine industry (3, 4). Further, unwarranted quarantine of horses with EP can lead to restrictions in import and export trade of horses or their participation in international equestrian sports (4–6).

Disease transmission, epidemiology, severity and drug susceptibility of EP can vary greatly depending on whether infected by *B. caballi* and/or *T. equi*. Therefore, accurate parasite identification is important. Direct detection, which involves microscopic examination, blood smear staining, lymphatic tissue puncture and histological examination of lymph nodes (7), relies on the experience and proficiency of the examiner to determine parasite sensitivity and specificity. It also relies on parasitemia being high, which can mean less sensitive in the persistent stage of infection when parasitemia levels are generally very low (8, 9). Immunological diagnostic methods such as enzyme-linked immunosorbent assay (ELISA), complement fixation test (CFT), and indirect fluorescent antibody test (IFAT) are often used to diagnose carrier horses. Nowadays, there are many recombinant proteins used for ELISA diagnosis of EP. Among them, *T. equi* (*EMA-1, EMA-2*) and *B. caballi* (*RAP-1, Bc48*) which can be expressed in *E. coli*, yield good experimental results (10–13). CFT can accurately diagnose early infections with good sensitivity and specificity, but cannot be detected in individuals who have undergone drug treatment or have developed anti-complement reactions. IFAT is one of the methods recommended by World Organization for Animal Health and readily used in the diagnosis of clinical hosts with EP. However, the technique can exhibit antibody detection limitations and cross-reactivity issues, which can cause misdiagnosis.

Nucleic acid detection methods for *B. caballi* and *T. equi*, including nested PCR (nPCR), loop-mediated isothermal amplification and quantitative PCR (qPCR) (12, 14–16). These methods are based on amplifying specific sequences of *T. equi and B. caballi* and exhibit greater sensitivity than microscopic examination (17–19). Nested PCR is widely used to diagnose sub-clinical infections and help control the importation of infected animals and provide advice for medical treatment (19). Mahmoud et al. analyzed 139 equid blood samples for EP using nPCR and IFAT, respectively (17). IFAT positively identified EP in 48.9%, while nPCR positively identified 56.8%. Nardini et al. conducted a comparison between nPCR and another three PCR-based methods for the detection of EP in 103 field samples (20). Quantitative PCR exhibits high specificity, sensitivity and stability, and is widely used in the qualitative and quantitative detection of various pathogens. The target qPCR genomic site for EP include the *EMA-1* gene of *T. equi* (21, 22) and the 48 kDa merozoite rhoptry protein (*BC48*) gene of *B. caballi* (23) or the *18S rRNA* gene of *T. equi* and *B. caballi* (24–26). Lobanov et al. designed a duplex real-time quantitative PCR (duplex qPCR) method for the *T. equi EMA-1* and *B. caballi 18S rRNA* genes (22). They verified the method using 362 samples of cELISA-negative horses and detected no false positives. Despite such progress in this field, there still remains a need for faster and more sensitive diagnostic methods for EP. Therefore, in this study, two techniques of nPCR and duplex fluorescence qPCR that could simultaneously detect *T. equi* and *B. caballi* in the diagnosis of EP were developed and compared.

## Materials and Methods

### Animals

The present study was conducted in April during a period of high incidence of equine piroplasmosis in 2019 at Ulan Butong Grassland of Chifeng City, Inner Mongolia Autonomous Region, China. Thirty-six tick-bitten malnourished Mongolia horses (4 stallions, 14 geldings and 18 mares) aged 6.7 ± 1.5 years and with mean live weight 286.5 ± 21.3 kg were used for the study. The general condition of horses were fully monitored by routine clinical examinations. The body condition score of horses ranged between 3 and 4, scored on a 9-point scale (1–9, where 1 represents lean minus and 9 represents fat plus), according to the guidelines described by Henneke et al. (27).

### Blood Sample and DNA Collection

Blood samples were obtained from the jugular vein of the horses into 10 mL vacuum tubes containing ethylenediaminetetraacetic acid as an anticoagulant (BD Vacutainer, USA). All blood samples were stored in 4 °C and centrifuged for 10 min at 3,000 rpm at 4 °C to separate the red blood cells, and then kept frozen at −80 °C for further analyses.

DNA from 200 uL of red blood cells was extracted. The extraction was carried out using TaKaRa MiniBEST Universal Genomic DNA Extraction Kit Version 5.0 (Takara Biomedical Technology Co., Ltd, Dalian, China) according to the instructions. Standard negative blood samples were obtained from EP-free horses which were detected and confirmed negative using the method described in a previous study (28). *Theileria sinensis* DNA samples were donated by Heilongjiang Bayi Agricultural University.

### Standard Positive Plasmid Preparation

*18S rRNA* coding sequences (Z15104.1 and Z15105.1) of *B. caballi* and *T. equi* (according to the sequence information published by GenBank) were synthesized using Nanjing Kingsley Biotechnology Co., Ltd., and cloned them into pUC57 vector, which were named pUC57-*T. equi* and pUC57-*B. caballi*, respectively.

### Primers Design and Nested PCR

Based on the *18S rRNA* gene sequence of *T. equi* (Z15105.1, DQ287951.1, EU642511.1, KM046918.1, KF559357.1, AY150063.2) and *B. caballi* (KY952238.1, KY952236.1, KY952233.1, AY309955.1, AY534883.1, Z15104.1) from GenBank, a set of primers were designed from the conserved regions shared by all gene sequences (Supplementary Figure 1) using Primer 5.0. Table 1 lists the designed internal and external nPCR primers.

**Table 1:** Primer sequences of nested PCR for detecting *T. equi* and *B. caballi*.

	**Primer name**	**Sequence (5^**′**^–3^**′**^)**	**Amplicon Size (T/B bp)^**a**^**	**Product length (T/B bp)^**a**^**
First nested PCR primers outer	18S Out - F	CACATCTAAGGAAGGCAGCA	396/372	1016/986
	18S Out - R	CAGGACATCTAAGGGCATCA	1411/1357	
Second nested PCR primers inner	18S In - F	TTGGAGGGCAAGTCTGGT	530/505	368/341
	18S In - R	TTTCGCAGTAGTTCGTCTTT	897/845	

a *“T” = T. equi; “B” = B. caballi*.

The first round of nPCR was conducted in a 20 μL reaction system containing 10 μL of 2 × PCR Master Mix (Beyotime Biotechnology, Shanghai, China), 0.5 μL of each primer (18S Out – F, 10 μM; 18S Out – R, 10 μM), and 1 ng of a standard positive plasmid. A pre-denaturation step at 95 °C for 5 min was conducted and a total of 35 amplification cycles was performed. Each cycle consisted of denaturation at 95 °C for 15 s, annealing at 56 °C for 15 s, and extension at 72 °C for 30 s. A final extension was also included at 72 °C for 7 min in the PCR profile. In total 0.5 μL of the amplified products were used in the second round of the nPCR, also in a 20 μL reaction system, changing the primers to 18S In – F (10 μM) and 18S In – R (10 μM). The PCR reaction conditions and programs were same as in round one. The amplified products were electrophoresed in 4% agarose gels and the results were documented using the gel doc-IT^2^ imager system (UVP Inc, CA, USA).

### Specificity and Sensitivity of Nested PCR

To determine detection specificity of nPCR, known standard positive plasmids were used for amplification, negative blood DNA samples and genomic DNA of *T. sinensis* were used as negative controls, and ddH_2_O was used as a blank control. To determine detection sensitivity, the standard positive plasmid was diluted into different concentrations (1 × 10^−1^ to 1 × 10^−8^ ng/μL) and nPCR amplification were performed. The lowest detectable concentration was determined by observing agarose gel electrophoresis results of second round PCR products.

### Detection of Horse Blood Samples by Nested PCR

Using nPCR, 36 horse blood samples were tested. The detection method was performed in triplicate, and second round PCR products were sequenced at Sangon Biotech Co. Ltd., Shanghai. Further, the same set of samples was also detected by a multinested PCR (mnPCR) targeting the *EMA-1* gene of *T. equi* and the *RAP-1* gene of *B. caballi* as previously described (15) and the primers were shown in Table 2.

**Table 2:** Primer sequences of multinested PCR for detecting *T. equi* and *B. caballi*.

	**Target species**	**Gene**	**Primer name**	**Sequence 5^**′**^–3^**′**^**	**Amplicon Size (bp)**	**Reference**
First multi-nested PCR primers outer	*T. equi*	EMA-1	EMA1-outer-R	TGTCGTCACTTAGTAAAATAGAG	828	Montes Cortés et al. (15)
			EMA1-outer-F	ATGATTTCCAAATCCTTTGC		
	*B. caballi*	RAP-1	RAP1-outer-R	GCTTCATGTACCACTTCTTATAC	891	
			RAP1-outer-F	GCGCCCTCTTGCTYGTAG		
Second multi-nested PCR primers inner	*T. equi*	EMA-1	EMA1-inner-R	TGTCCTTGATGTGCCTGAC	274	Montes Cortés et al. (15)
			EMA1-outer-F	ATGATTTCCAAATCCTTTGC		
	*B. caballi*	RAP-1	RAP-1outer-R	GCTTCATGTACCACTTCTTATAC	566	
			RAP1-inner-F	GTACCAACCGCTGACCCTTC		

### Optimization of Primers and Probes for Duplex Real-Time Fluorescence Quantitative PCR

Table 3 lists the primer sequences and probes used in this study. Primers and probes were designed from the species-specific region of the 18S rDNA (Supplementary Figure 1), using Primer Express (version 3.0) and Primer (version 5.0) software. TaqMan probes with reporter dye 6-Carboxyfluorescein (FAM) or 5-Hexachloro-fluorescein (HEX) at the 5' end, and Black Hole Quencher-1(BHQ1) at the 3' end were labeled.

**Table 3:** Sequence of primers and probes of duplex real-time fluorescence quantitative PCR for detecting *T. equi* and *B. caballi*.

**Primer and probes name**	**Sequence (5^**′**^–3^**′**^)**
*B. caballi*-F	ACCTGCTAACTAGCTTCCCTTT
*B. caballi*-R	ACCTTTCGGAGCAGGAAAA
*B. caballi*-Probe	FAM-ACAGCTTGTCGCTGTAAAGTCCCTCT-BHQ1
*T. equi*-F	AATTTCTGCTGTTTCGTTGACT
*T. equi-R*	TCTGGCTCCTAAAACCAACA
*T. equi*-Probe	HEX-ACCCAACCAAGCCGCAACGA-BHQ1

### Specificity and Sensitivity of Duplex Real-Time Fluorescence Quantitative PCR

To determine detection specificity of duplex fluorescence qPCR, a 50 μL reaction volume was used, containing 1 μL (20 pmol) of each forward and reverse primer, 0.5 μL (10 pmol) of TaqMan probes, 0.5 μL (1 × 10^−1^ ng/μL) of standard positive plasmid, 25 μL of *Taq*Man™ Real-Time PCR Master Mixes (Thermo Fisher Scientific, MA, USA), and ddH_2_O to 50 μL. The following thermocycling program was used: 40–45 cycles of 95 °C for 15 s, 60 °C for 15 s, and 72 °C for 30 s with an initial cycle of 95 °C for 30 s, using the Roche LightCycler 480 real time PCR system (Hoffmann-La Roche Ltd, Basel, Schweiz). All amplifications and detections were conducted using a 96-well reaction plate with optical caps (Sangon Biotech, Shanghai, China). At each cycle, accumulation of PCR products were detected by monitoring the increase in reporter dye fluorescence of the TaqMan probes. To test sensitivity, the standard positive plasmid was diluted into different concentrations (1 × 10^−1^ to 1 × 10^−8^ ng/μL) and the lowest detection efficiency and number of copies were determined.

### Detection of Horse Blood Samples by Duplex Real-Time Fluorescence Quantitative PCR

The 36 horse blood samples consisted with those detected by nPCR were tested using duplex fluorescence qPCR, and were performed in triplicate.

## Results

### PCR Results of Standard Positive Plasmids

**Figure 1:**
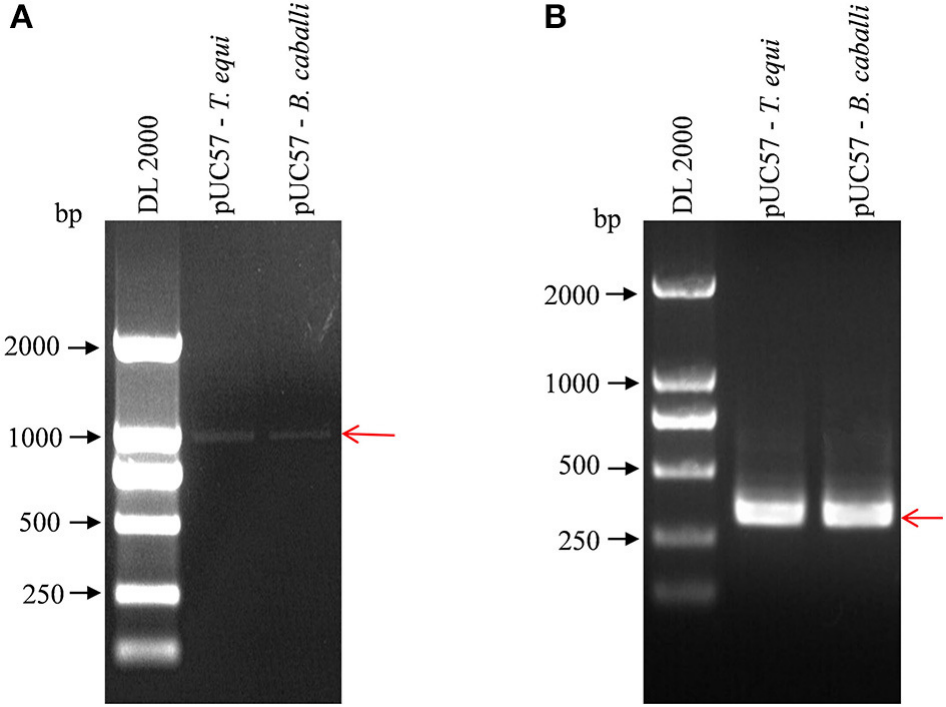
Detection of pUC57-*T. equi* and pUC57-*B. caballi* by nested PCR. **(A)** First round PCR product amplified with 18S Out – F primer and 18S Out – R primer. **(B)** Second round PCR product amplified with 18S In – F primer and 18S In – R primer. All PCR products were electrophoresed on 1% agarose gels and stained with ethidium bromide.

The nPCR products were analyzed of standard positive plasmids using agarose gel electrophoresis. First round amplification products of pUC57-*T. equi* and pUC57-*B. caballi* (using 18S Out primers) were about 1,000 bp in length, demonstrated by clear and complete electrophoretic bands of the amplified products (Figure 1A). Then, first-round amplification products were amplified a second time (with 18S In primers), revealing clear and bright electrophoretic bands at about 400 bp (Figure 1B).

Fragment lengths obtained from the amplification were consistent with the target product, indicating successful primer design. For further verification, the amplified products were sent to Shanghai Biotechnology Co., Ltd. for sequencing, which confirmed correct sequences. Therefore, the designed primers could be effectively used in subsequent experiments.

### Specificity and Sensitivity of Nested PCR

**Figure 2:**
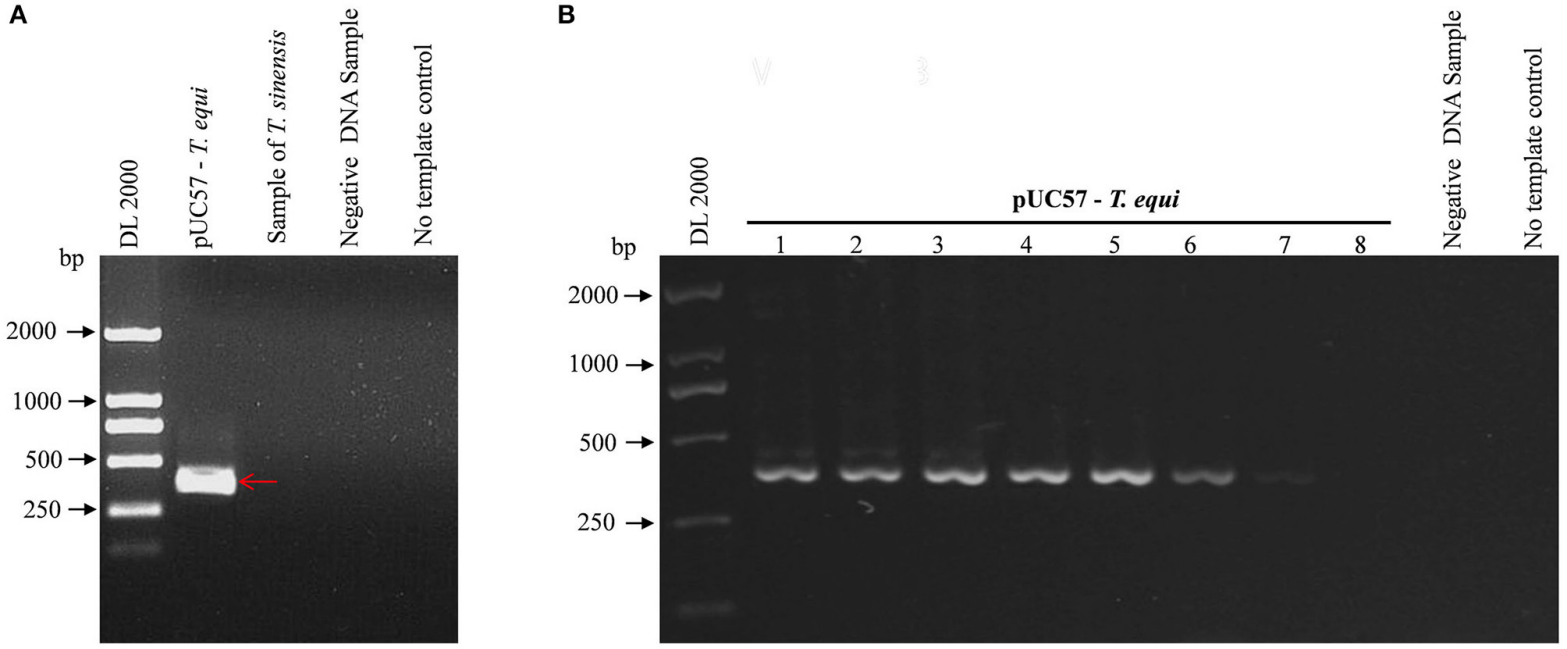
Specificity and sensitivity of pUC57-*T. equi* detected by nested PCR. **(A)** Specificity of the nested PCR. *T. sinensis* is unrelated DNA and used as a control. **(B)** Sensitivity of the nested PCR. Lane 1-8, this experiment was carried out using different concentrations of pUC57-*T. equi* DNA (ng): 1 × 10^−1^, 1 × 10^−2^, 1 × 10^−3^,1 × 10^−4^, 1 × 10^−5^, 1 × 10^−6^, 1 × 10^−7^, and 1 × 10^−8^, respectively. All PCR products were electrophoresed on 1% agarose gels and stained with ethidium bromide.

Figure 1 shows a single band at the expected sizes following nPCR, indicating successful primer design. Moreover, Figure 2A shows the absence of a target band when primers were used with *T. sinensis*, suggesting primers are specific for EP detection. nPCR results, however, failed to easily distinguish the two *T. equi* and *B. caballi* pathogens by electrophoresis alone. To determine nPCR sensitivity, a 10-fold serial dilution of the standard positive plasmid (from an initial concentration of 1 ng/μL) was used. Using this method, standard positive plasmids at 1 × 10^−1^ to 1 × 10^−7^ ng/μL could be detected (Figure 2B), indicating that the nPCR method has high sensitivity and can amplify DNA concentrations as low as 1 × 10^−7^ ng/μL.

### Nested PCR and Multinested-PCR of Horse Blood Samples

Using nPCR, analysis of the 36 blood samples revealed 29 positive samples, demonstrating a positivity rate of 80.56%. Of the 29 positive samples, 21 were infected with *T. equi*, six with *B. caballi*, and two were mixed (Supplementary Figure 2, Table 4). This analysis was performed in triplicate, and showed the same results. Sequencing analysis of the nPCR products confirmed these results which showed that six were positive for *B. caballi* and 21 for *T. equi*, suggesting that nPCR can be an effective tool in detecting EP infection in clinical disease. However, to separate the 27 bp difference in PCR product of DNA from horse blood samples simultaneous infected with *T. equi* and *B. caballi*, electrophoresis with 4% agarose gels was needed. The mnPCR for the 36 blood samples showed that 22.22% of the blood samples (8/36) were positive for equine piroplasmids. The prevalence of infection for *T. equi* was 2.78% (1/36), and 19.44% (7/36) for *B. caballi* (Supplementary Figure 3, Table 4). The results mean that nPCR method is more sensitive when the parasitemia was low.

**Table 4:** Comparison of nested PCR, multi-nested PCR and duplex real-time fluorescence quantitative PCR for detecting equine piroplasmosis^a^.

**Clinical samples**	**Value of A260/A280 of DNA**	**Quantitative PCR** ^**b**^		**Nested PCR**		**Sequencing** ^**c**^		**Multi-nested PCR**	
	**from clinical samples**	
		***B. caballi***	***T. equi***	***B. caballi***	***T. equi***	***B. caballi***	***T. equi***	***B. caballi***	***T. equi***
Sample 1	1.82	−	+	−	+	−	+	−	−
Sample 2	1.83	−	+	−	+	−	+	−	−
Sample 3	1.88	−	−	+	−	+	−	+	−
Sample 4	1.81	−	−	−	+	−	+	−	−
Sample 5	1.82	−	−	−	+	−	+	−	−
Sample 6	1.83	−	+	−	+	−	+	−	−
Sample 7	1.86	−	+	−	+	−	+	−	−
Sample 8	1.84	−	−	−	+	−	+	−	−
Sample 9	1.85	−	−	−	+	−	+	−	−
Sample 10	1.86	−	−	−	+	−	+	−	−
Sample 11	1.85	−	+	−	+	−	+	−	−
Sample 12	1.85	−	−	−	+	−	+	−	−
Sample 13	1.85	−	−	−	+	−	+	−	−
Sample 14	1.81	−	−	−	+	−	+	−	−
Sample 15	1.84	−	+	−	+	−	+	−	+
Sample 16	1.83	−	−	+	−	+	−	+	−
Sample 17	1.81	−	+	−	+	−	+	−	−
Sample 18	1.95	−	+	−	+	−	+	−	−
Sample 19	1.95	+	−	+	−	+	−	+	−
Sample 20	1.84	−	−	−	−	−	−	−	−
Sample 21	2.01	−	−	−	+	−	+	−	−
Sample 22	1.84	−	−	−	−	−	−	−	−
Sample 23	1.82	+	+	+	+	+	+	+	−
Sample 24	1.85	−	−	−	−	−	−	−	−
Sample 25	1.8	−	−	+	−	+	−	+	−
Sample 26	1.83	−	−	−	−	−	−	−	−
Sample 27	1.93	−	+	−	+	−	+	−	−
Sample 28	1.84	−	−	−	−	−	−	−	−
Sample 29	1.82	−	−	+	−	+	−	+	−
Sample 30	1.84	−	+	−	+	−	+	−	−
Sample 31	1.99	−	−	−	+	−	+	−	−
Sample 32	1.8	−	+	−	+	−	+	−	−
Sample 33	1.82	−	+	+	+	+	+	−	−
Sample 34	1.8	−	−	+	−	+	−	+	−
Sample 35	1.8	−	−	−	−	−	−	−	−
Sample 36	1.83	−	−	−	−	−	−	−	−

a *“+” = positive; “-” = negative*.

b *Quantitative PCR = duplex real-time fluorescence quantitative PCR*.

c *Sequencing results mean the results of second round PCR products in nested PCR*.

### Specificity of Duplex Real-Time Fluorescence Quantitative PCR

**Figure 3:**
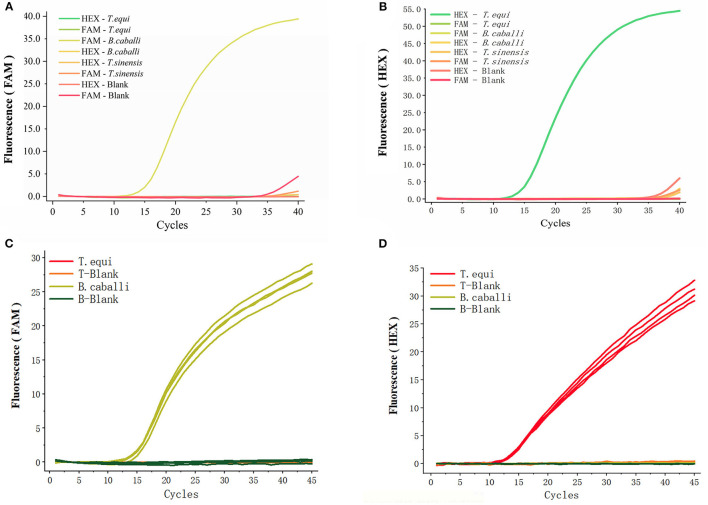
Specificity of the primers and probes for duplex real-time fluorescence quantitative PCR. **(A,B)** obtained from FAM panel and HEX panel, respectively. Various probes and templates were added to the reaction system. *T. sinensis* is unrelated DNA and used as a control. **(C,D)** Two different types of primers and probes were added into one reaction and there were four repeated samples, only the template was different.

An array of fluorescent probes and templates (Figures 3A,B) were initially tried, before deciding on HEX to label *T. equi* (using a detection wavelength range of 533–580 nm) and FAM to label *B. caballi* (using a detection wavelength range of 465–510 nm). Only a FAM-*B. caballi* amplification curve in the FAM panel (Figure 3A), and a HEX-*T. equi* amplification curve in the HEX panel were detected (Figure 3B).

The two different types of primers and probes were then added into one reaction vessel, changing only the template (Figures 3C,D). An amplification curve in the FAM panel was observed when *B. caballi* template was used (Figure 3C), and an amplification curve in the HEX panel when *T. equi* template was used (Figure 3D). These results indicate that the duplex fluorescence qPCR method demonstrates good specificity.

### Sensitivity of Duplex Real-Time Fluorescence Quantitative PCR

**Figure 4:**
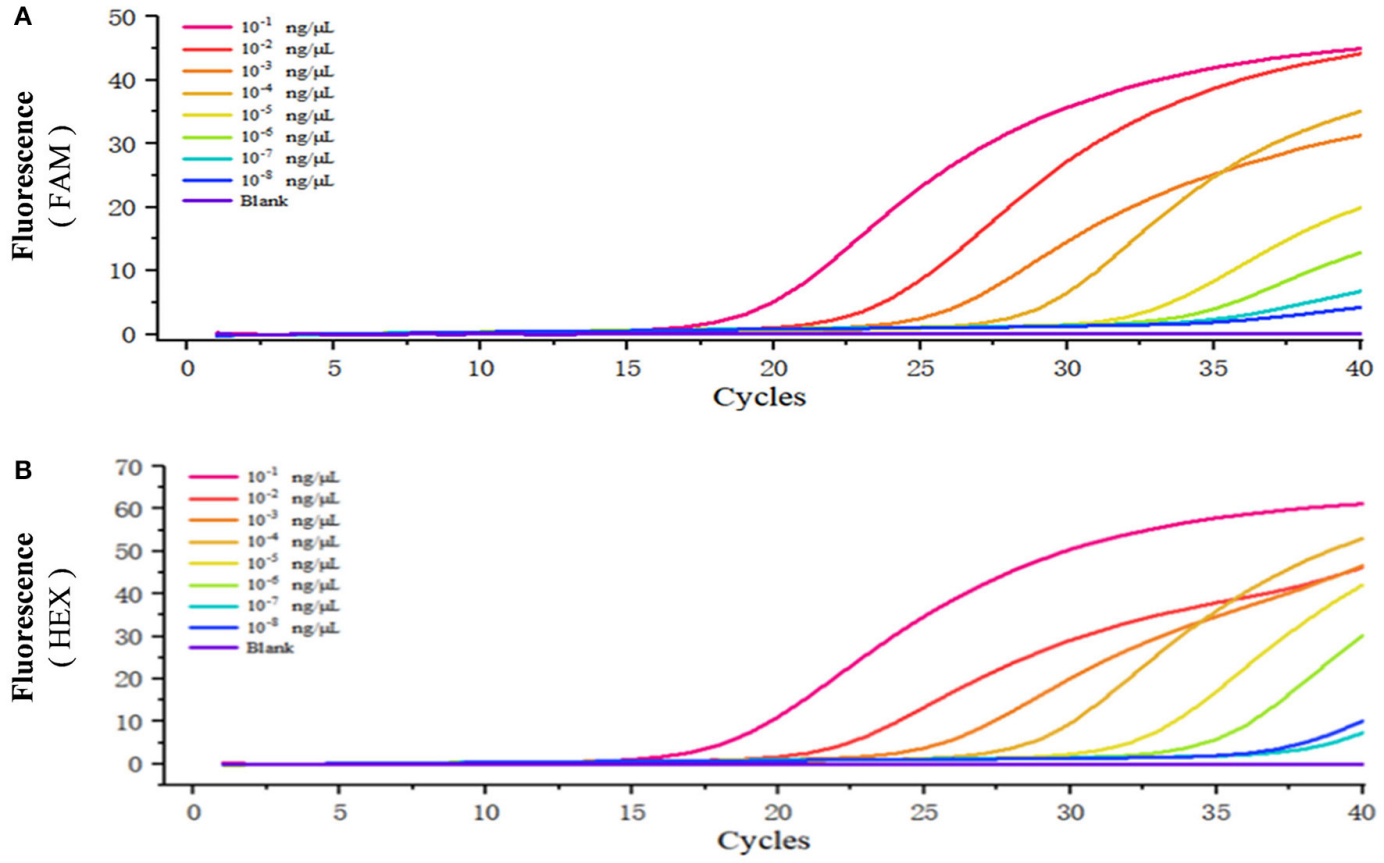
Sensitivity of the duplex real-time fluorescence quantitative PCR method. The standard positive plasmid was diluted into different concentrations (ng/μL): 1 × 10^−1^, 1 × 10^−2^, 1 × 10^−3^, 1 × 10^−4^, 1 × 10^−5^, 1 × 10^−6^, 1 × 10^−7^, and 1 × 10^−8^, respectively, and sterile ddH_2_O was considered as blank control. **(A)** results of the standard positive plasmid of *B. caballi*. **(B)** results of the standard positive plasmid of *T. equi*.

**Figure 5:**
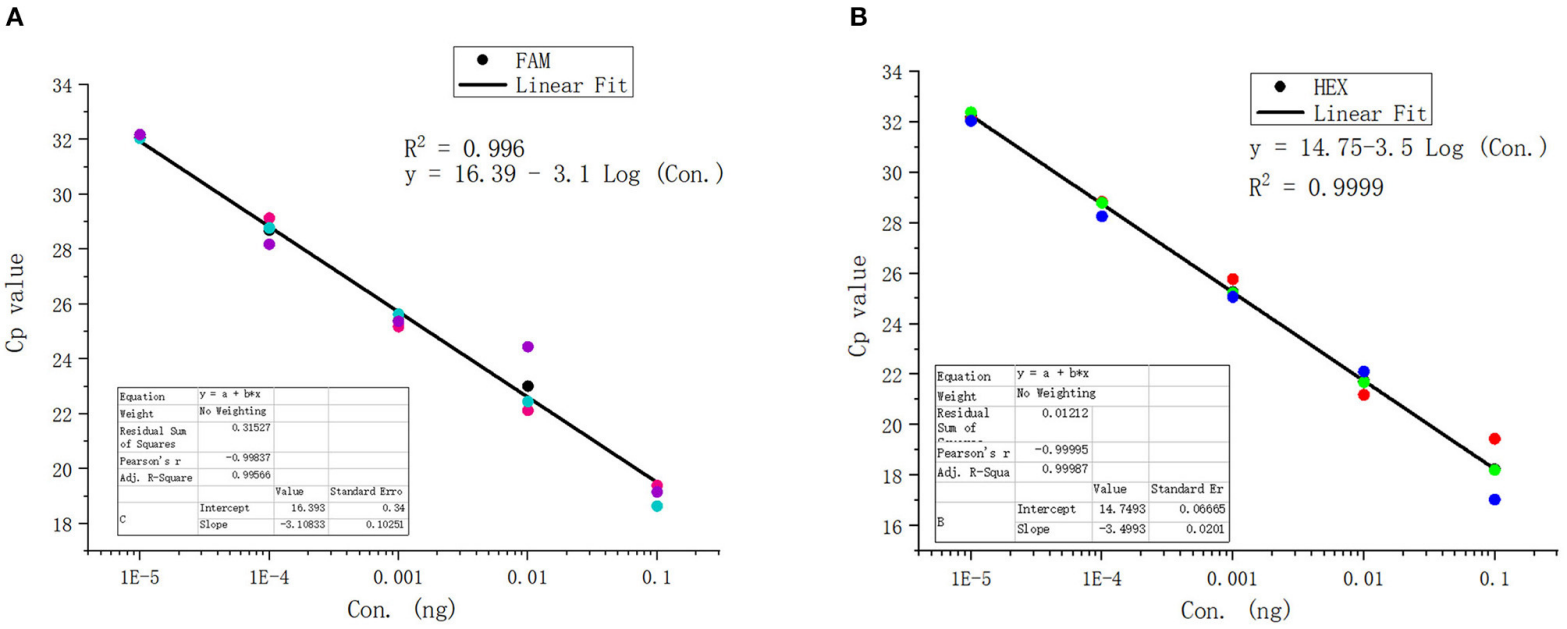
Standard curve of the duplex real-time fluorescence quantitative PCR method. **(A)** results of the standard positive plasmid of *B. caballi*. **(B)** results of the standard positive plasmid of *T. equi*.

The standard positive plasmid template was diluted into different concentrations (ng/μL): 1 × 10^−1^, 1 × 10^−2^, 1 × 10^−3^,1 × 10^−4^, 1 × 10^−5^, 1 × 10^−6^, 1 × 10^−7^, and 1 × 10^−8^, respectively, using 1 μL of template in each reaction system and sterile ddH_2_O as a blank control, and assayed the samples using duplex fluorescence qPCR. In the absence of a template, we failed to observe an amplification curve in either FAM or HEX panel (Figures 4A,B). When 1 × 10^−7^ and 1 × 10^−8^ ng/μL of template was used, we observed a weak amplification curve (Figures 4A,B). Figure 5 shows standard curves generated from combined data of these runs. The correlation coefficient of *B. caballi* was R^2^ = 0.996, and the standard curve equation was y = 16.39–3.1 log(x) (Figure 5A). The correlation coefficient of *T. equi* was R^2^ = 0.999, and the standard curve equation was y = 14.75–3.5 log(x) (Figure 5B). These results demonstrate that duplex fluorescence qPCR detected a minimum of 4.47 × 10^2^ copies/μL, indicating that it can identify pathogenic EP species with high efficiency.

### Duplex Real-Time Fluorescence Quantitative PCR of Horse Blood Samples

Using duplex fluorescence qPCR, analysis of the 36 blood samples revealed 14 infected samples (shown in Supplementary Figure 4, demonstrating a positivity rate of 38.89%. Of the 14 positive samples, 12 were infected with *T. equi*, one with *B. caballi*, and one was mixed (Table 4). Then, 36 samples were tested in triplicate using this method and the results were the same. Compared with the nPCR results, duplex fluorescence qPCR demonstrates lower sensitivity.

## Discussion

Rapid and accurate identification of pathogens is the basis of animal disease control. In this study, 36 blood samples from EP clinically suspect horses were analyzed individually for *T. equi* and *B. caballi*, and a nPCR and a duplex fluorescence qPCR assay for simultaneous detection of both causative agents of EP were developed. We aimed to provide a convenient and economical PCR-based method for the diagnosis, epidemiological investigation, prevention and control of EP in clinical settings.

Nowadays, many methods have been developed for clinical diagnosis and detection of EP (5, 7, 29). Owing to its simple operation, low cost, convenience and fast detection, blood smear staining has remained the standard method in clinical EP diagnosis. However, the method requires skilled and experienced operators to distinguish mixed from isolated infections. Moreover, the technique cannot easily detect parasites when parasitemia is low, increasing the possibility of misdiagnosis (19). Serological diagnostic methods, such as CFT and IFAT, have been widely used in clinical and laboratory detection of EP (7, 14, 30). However, the preparation of recombinant proteins required by these methods is complex and the operation process can be complicated.

Biomolecular detection methods have developed rapidly in recent years. Particularly, owing to its high sensitivity and specificity, PCR technology has been established as the best method for detecting and differentiate EP (15, 26, 31). Previous studies have shown that nPCR is more sensitive and specific than traditional PCR in detecting EP (9, 16, 19). Nicolaiewsky et al. developed a nPCR for the detection of *T. equi* based on the merozoite antigen *EMA-1* gene sequence (9). Other studies used a nPCR method based on *EMA-1* and *18S rRNA or BC48* gene for the detection of *T. equ*i and *B. caballi*, respectively (17, 20). In this study, we established a nPCR method, based on the homologous region of *18S rRNA* gene sequence of *T. equi* and *B. caballi*, which could specifically and simultaneously detect EP. In addition, Montes Cortés et al. developed a mnPCR for simultaneous detection of the equine piroplasmids *T. equi* and *B. caballi*, by amplification of five genetic markers (15). The present study revealed that the nPCR was more sensitive than mnPCR targeting the *EMA-1* gene of *T. equi* and the *RAP-1* gene of *B. caballi* when the parasitemia was low. Herein, nPCR is convenient with a low cost ($0.8 per sample) and takes about 3 h. However, this method required a 4% gel to separate the 27 bp difference in PCR product.

In the present study, we also established a duplex real-time fluorescence quantitative PCR method that only takes about 1h and costs less than $4, which used single-target *18S rRNA* gene for the simultaneous detection of *T. equi* and *B. caballi*. In contrast to Lobanov et al., who used a duplex real-time qPCR method based on two different genes (*EMA-1* and *18S rRNA*) for the detection of both causative agents of EP (22), we designed and synthesized a pair of specific primers and fluorescent probes according to the *18S rRNA* sequence of *T. equi* and *B. caballi*, respectively. We labeled the fluorescent probes corresponding to *T. equi* with HEX fluorescein, and the fluorescent probes corresponding to *B. caballi* with FAM fluorescein. By using two fluorescent probes and two pairs of primers simultaneously in a reaction system, this method could successfully detect and distinguish the two pathogens of EP. Unfortunately, the method failed to detect at low parasitemia levels, which may be because most of the genomic DNA remained contained in the horse blood DNA sample, and only one round of PCR was used. Importantly, both methods revealed PCR products consistent with DNA sequencing results.

## Conclusion

Nested PCR method detected *T. equi* and *B. caballi* positive samples and mixed samples with good specificity and sensitivity, it is really a convenient and economical method, especially for some economically underdeveloped countries. Meanwhile, our duplex fluorescence qPCR detection method successfully detected and distinguished *T. equi* and *B. caballi*, although with low sensitivity, as it could only identify the two parasite strains when parasitemia was high. Altogether, these methods have potential to provide effective technical support for the diagnosis, epidemiological investigation, prevention and control of EP in clinical settings.

## Data Availability Statement

The original contributions presented in the study are included in the article/Supplementary Material, further inquiries can be directed to the corresponding author/s.

## Ethics Statement

The animal study was reviewed and approved by The Ethical Committee of Shenyang Agricultural University approved the laboratory animal experiments (Permit No. SYXK2011-0001). Written informed consent was obtained from the owners for the participation of their animals in this study.

## Author Contributions

KL and YZ performed the experiments. YY participated in data analysis. ZL helped with sampling and experiments. LD and YZ edited the manuscript. All authors read and approved the final manuscript.

## Funding

This study was supported by CAMS Innovation Fund for Medical Sciences (CIFMS) (Grant No. 2019-I2M-5-042) and the Foundation of Shenyang Science and Technology Transformation Project (Grant No. 21-116-3-39).

## Conflict of Interest

The authors declare that the research was conducted in the absence of any commercial or financial relationships that could be construed as a potential conflict of interest.

## Publisher's Note

All claims expressed in this article are solely those of the authors and do not necessarily represent those of their affiliated organizations, or those of the publisher, the editors and the reviewers. Any product that may be evaluated in this article, or claim that may be made by its manufacturer, is not guaranteed or endorsed by the publisher.

## References

[1] Guidi E Pradier S Lebert I Leblond A. Piroplasmosis in an endemic area: analysis of the risk factors and their implications in the control of Theileriosis and Babesiosis in horses. Parasitol Res. (2015) 114:71–83. 10.1007/s00436-014-4161-9 25280516

[2] Knowles DP Kappmeyer LS Haney D Herndon DR Fry LM Munro JB. Discovery of a novel species, *Theileria haneyi* n. sp, infective to equids, highlights exceptional genomic diversity within the genus Theileria: Implications for apicomplexan parasite surveillance. Int J Parasitol. (2018) 48:679–90. 10.1016/j.ijpara.2018.03.010 29885436

[3] Elelu N. Tick-borne relapsing fever as a potential veterinary medical problem. Vet Med Sci. (2018) 4:271–9. 10.1002/vms3.108 29943903 PMC6236141

[4] Short MA Clark CK Harvey JW Wenzlow N Hawkins IK Allred DR. Outbreak of equine piroplasmosis in Florida. J Am Vet Med Assoc. (2012) 240:588–95. 10.2460/javma.240.5.588 22332629

[5] Tirosh-Levy S Gottlieb Y Fry LM Knowles DP Steinman A. Twenty years of equine piroplasmosis research: global distribution, molecular diagnosis, and Phylogeny. Pathogens. (2020) 9:926. 10.3390/pathogens9110926 33171698 PMC7695325

[6] Kerber CE Ferreira F Pereira MC. Control of equine piroplasmosis in Brazil. Onderstepoort J Vet Res. (1999) 66:123–7. 10486829

[7] Lempereur L Beck R Fonseca I Marques C Duarte A Santos M. Guidelines for the detection of *Babesia* and *Theileria* parasites. Vector-Borne Zoonotic Dis. (2017) 17:51–65. 10.1089/vbz.2016.1955 28055573

[8] Bashiruddin JB Camma C Rebelo E. Molecular detection of *Babesia equi* and *Babesia caballi* in horse blood by PCR amplification of part of the 16S rRNA gene. Vet Parasitol. (1999) 84:75–83. 10.1016/S0304-4017(99)00049-7 10435792

[9] Nicolaiewsky TB Richter MF Lunge VR Cunha CW Delagostin O Ikuta N. Detection of *Babesia equi* (Laveran, 1901) by nested polymerase chain reaction. Vet Parasitol. (2001) 101:9–21. 10.1016/S0304-4017(01)00471-X 11587829

[10] Xuan X Larsen A Ikadai H Tanaka T Igarashi I Nagasawa H. Expression of *Babesia equi* merozoite antigen 1 in insect cells by recombinant baculovirus and evaluation of its diagnostic potential in an enzyme-linked immunosorbent assay. J Clin Microbiol. (2001) 39:705–9. 10.1128/JCM.39.2.705-709.2001 11158131 PMC87800

[11] Ueti MW Palmer GH Kappmeyer LS Scoles GA Knowles DP. Expression of equi merozoite antigen 2 during development of *Babesia equi* in the midgut and salivary gland of the vector tick *Boophilus microplus*. J Clin Microbiol. (2003) 41:5803–9. 10.1128/JCM.41.12.5803-5809.2003 14662988 PMC308990

[12] Bhoora R Quan M Zweygarth E Guthrie AJ Prinsloo SA Collins NE. Sequence heterogeneity in the gene encoding the rhoptry-associated protein-1 (RAP-1) of *Babesia caballi* isolates from South Africa. Vet Parasitol. (2010) 169:279–88. 10.1016/j.vetpar.2010.01.009 20138703

[13] Ikadai H Xuan X Igarashi I Tanaka S Kanemaru T Nagasawa H. Cloning and expression of a 48-kilodalton *Babesia caballi* merozoite rhoptry protein and potential use of the recombinant antigen in an enzyme-linked immunosorbent assay. J Clin Microbiol. (1999) 37:3475–80. 10.1128/JCM.37.11.3475-3480.1999 10523537 PMC85671

[14] Bruning A Phipps P Posnett E Canning EU. Monoclonal antibodies against *Babesia caballi* and *Babesia equi* and their application in serodiagnosis. Vet Parasitol. (1997) 68:11–26. 10.1016/S0304-4017(96)01074-6 9066047

[15] Montes Cortés MG Fernandez-Garcia JL Habela Martinez-Estellez MÁ. A multinested PCR for detection of the equine piroplasmids *Babesia caballi* and *Theileria equi*. Ticks Tick Borne Dis. (2019) 10:305–13. 10.1016/j.ttbdis.2018.11.008 30472099

[16] Alhassan A Pumidonming W Okamura M Hirata H Battsetseg B Fujisaki K. Development of a single-round and multiplex PCR method for the simultaneous detection of *Babesia caballi* and *Babesia equi* in horse blood. Vet Parasitol. (2005) 129:43–9. 10.1016/j.vetpar.2004.12.018 15817201

[17] Mahmoud MS El-Ezz NT Abdel-Shafy S Nassar SA Namaky AE Khalil WB. Assessment of *Theileria equi* and *Babesia caballi* infections in equine populations in Egypt by molecular, serological and hematological approaches. Parasite Vector. (2016) 9:260. 10.1186/s13071-016-1539-9 27146413 PMC4857240

[18] Malekifard F Tavassoli M Yakhchali M Darvishzadeh R. Detection of *Theileria equi* and *Babesia caballi* using microscopic and molecular methods in horses in suburb of Urmia, Iran. Vet Res Forum. (2014) 5:129–33. 25568706 PMC4279624

[19] Rampersad J Cesar E Campbell MD Samlal M Ammons D. A field evaluation of PCR for the routine detection of *Babesia equi* in horses. Vet Parasitol. (2003) 114:81–7. 10.1016/S0304-4017(03)00129-8 12781470

[20] Nardini R Bartolomé Del Pino LE Cersini A Manna G Viola MR Antognetti V. Comparison of PCR-based methods for the detection of *Babesia caballi* and *Theileria equi* in field samples collected in Central Italy. Parasitol Res. (2021) 120:2157–64. 10.1007/s00436-021-07153-4 33855619

[21] Bhoora R Quan M Matjila PT Zweygarth E Guthrie AJ Collins NE. Sequence heterogeneity in the equi merozoite antigen gene (*ema*-1) of *Theileria equi* and development of an *ema*-1-specific TaqMan MGB^TM^ assay for the detection of *T. equi*. Vet Parasitol. (2010) 172:33–45. 10.1016/j.vetpar.2010.04.025 20493635

[22] Lobanov VA Peckle M Massard CL Scandrett WB Gajadhar AA. Development and validation of a duplex real-time PCR assay for the diagnosis of equine piroplasmosis. Parasite Vector. (2018) 11:125. 10.1186/s13071-018-2751-6 29499748 PMC5834856

[23] Heim A Passos LM Ribeiro MF Costa-Júnior LM Bastos BV Cabral DD. Detection and molecular characterization of *Babesia caballi* and *Theileria equi* isolates from endemic areas of Brazil. Parasitol Res. (2007) 102:63–8. 10.1007/s00436-007-0726-1 17828553

[24] Kim CM Blanco LB Alhassan A Iseki H Yokoyama N Xuan X. Diagnostic real-time PCR assay for the quantitative detection of *Theileria equi* from equine blood samples. Vet Parasitol. (2008) 151:158–63. 10.1016/j.vetpar.2007.10.023 18077095

[25] Bhoora RV Pienaar R Cornelius F Josemans A Matthee O Marumo R. Multiplex hydrolysis-probe assay for the simultaneous detection of *Theileria equi* and *Babesia caballi* infections in equids. Vet Parasitol. (2018) 255:61–8. 10.1016/j.vetpar.2018.03.022 29773138

[26] Bhoora R Quan M Franssen L Butler CM Kolk JH Guthrie AJ. Development and evaluation of real-time PCR assays for the quantitative detection of *Babesia caballi* and *Theileria equi* infections in horses from South Africa. Vet Parasitol. (2010) 168:201–11. 10.1016/j.vetpar.2009.11.011 20031328

[27] Henneke DR Potter GD Kreider JL Yeates BF. Relationship between condition score, physical measurements and body fat percentage in mares. Equine Vet J. (1983) 15:371–2. 10.1111/j.2042-3306.1983.tb01826.x 6641685

[28] Lei R Wang X Zhang D Liu Y Chen Q Jiang N. Rapid isothermal duplex real-time recombinase polymerase amplification (RPA) assay for the diagnosis of equine piroplasmosis. Sci Rep. (2020) 10:4096. 10.1038/s41598-020-60997-1 32139744 PMC7058082

[29] Hirata H Xuan X Yokoyama N Nishikawa Y Fujisaki K Suzuki N. Identification of a specific antigenic region of the P82 protein of *Babesia equi* and its potential use in serodiagnosis. J Clin Microbiol. (2003) 41:547–51. 10.1128/JCM.41.2.547-551.2003 12574244 PMC149686

[30] Bose R Jorgensen WK Dalgliesh RJ Friedhoff KT Vos AJ. Current state and future trends in the diagnosis of *babesiosis*. Vet Parasitol. (1995) 57:61–74. 10.1016/0304-4017(94)03111-9 7597794

[31] Elsawy BM Nassar AM Alzan HF Bhoora RV Ozubek S Mahmoud MS. Rapid detection of equine piroplasms using multiplex PCR and first genetic characterization of *Theileria haneyi* in Egypt. Pathogens. (2021) 10:1414. 10.3390/pathogens10111414 34832570 PMC8620363

