# Supplementary material for: Development of Nested PCR and Duplex Real-Time Fluorescence Quantitative PCR Assay for the Simultaneous Detection of Theileria equi and Babesia caballi
Source: Front Vet Sci. 2022 May 18;9:873190. doi: 10.3389/fvets.2022.873190 (PMC9158504; doi:10.3389/fvets.2022.873190)
Supplement: Supplementary file 1 [file Presentation_1.pdf]

Supplementary Material

1 Supplementary Figures

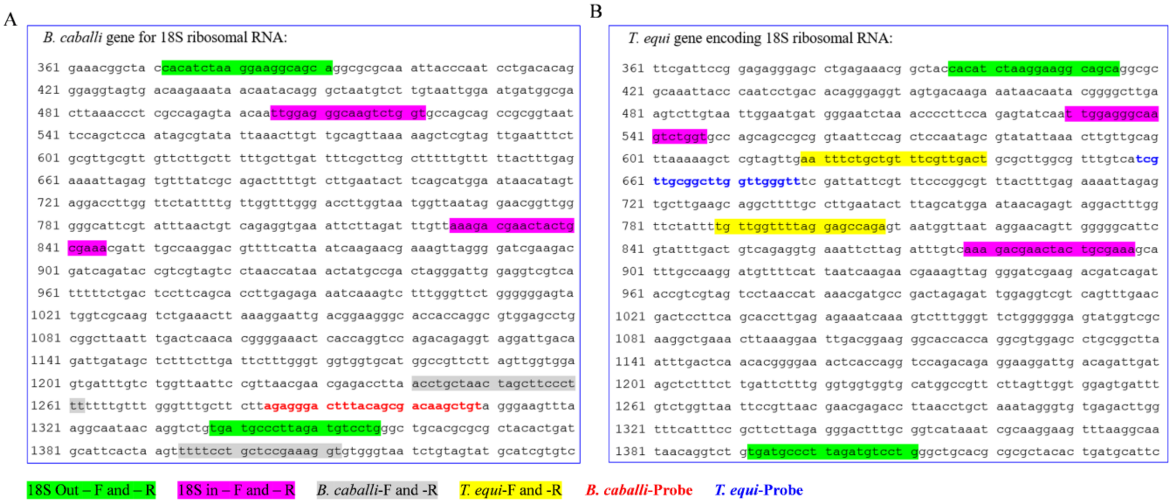

Supplementary Figure 1. Position of primers for nested PCR and duplex real-time fluorescence quantitative PCR. (A) primers for *B. caballi*. (B) primers for *T. equi*.

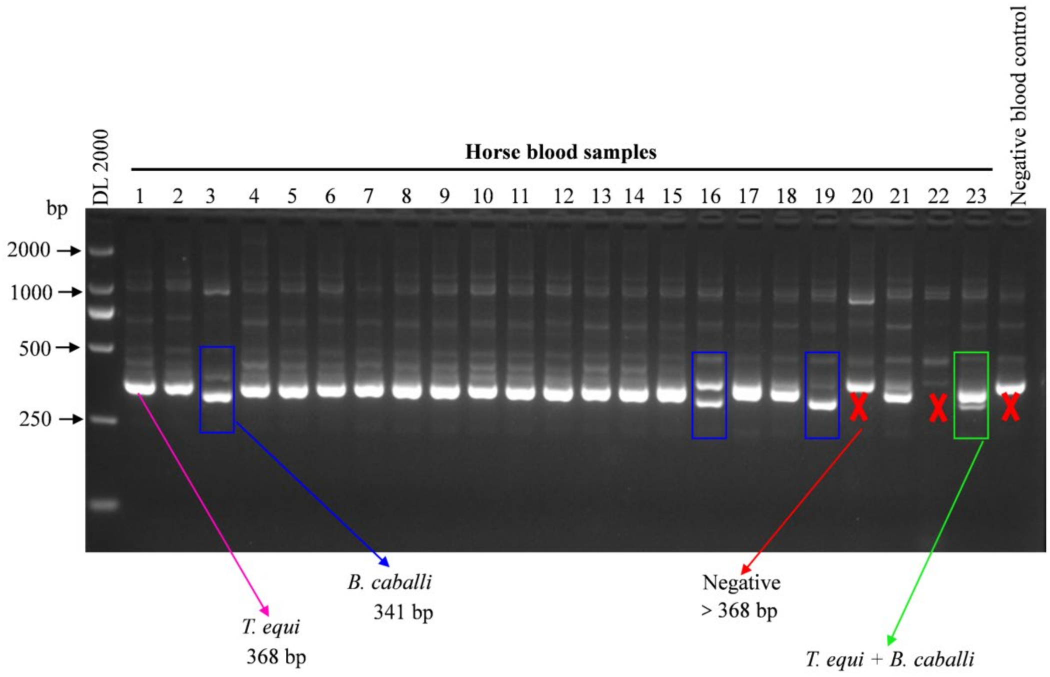

Supplementary Figure 2. DNA detection of some of the equine blood samples using nested PCR. All PCR products were electrophoresed on 4% agarose gels and stained with ethidium bromide. Products with no markers were from samples infected with *T. equi*. Products marked in blue squares were from samples infected with *B. caballi*. Products marked in green squares were from samples infected with both *T. equi* and *B. caballi*. Products marked in red crosses were from uninfected samples.

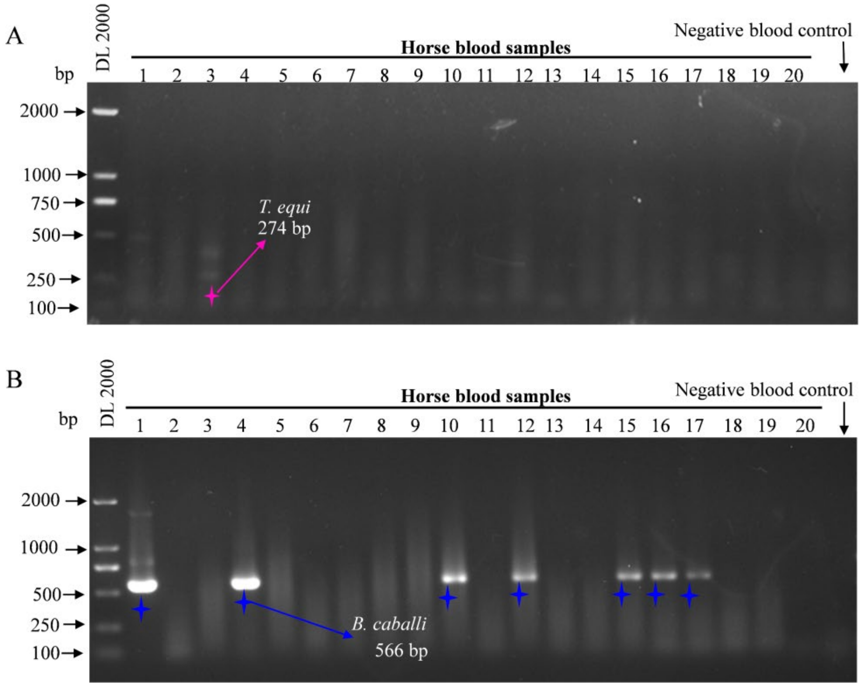

Supplementary Figure 3. DNA detection of some of the equine blood samples using multinested PCR. All PCR products were electrophoresed on 1.5% agarose gels and stained with ethidium bromide. (A) multinested PCR results for *T. equi*. (B) multinested PCR results for *B. caballi*.

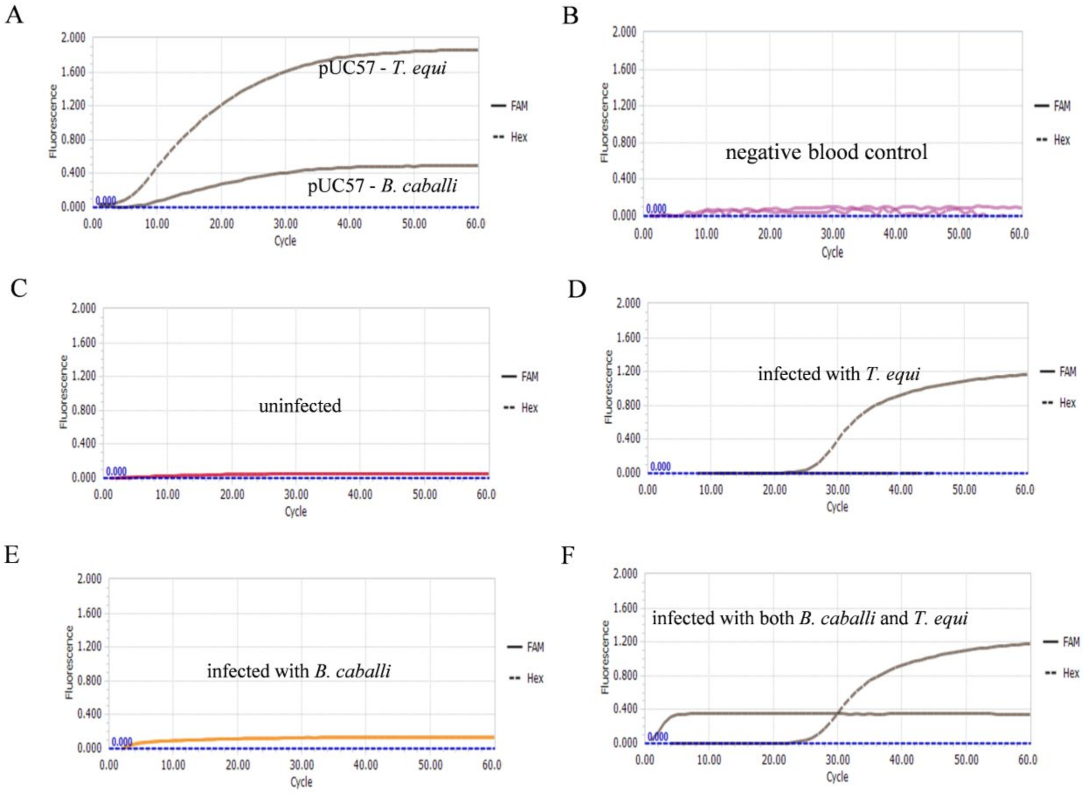

Supplementary Figure 4. DNA detection of some of the equine blood DNA using duplex real-time fluorescence quantitative PCR. (A) results of the standard positive plasmid of *T. equi* and *B. caballi*, (B) negative blood control, (C) uninfected sample, (D) infected with *T. equi*, (E) infected with *B. caballi*, (F) infected with both *T. equi* and *B. caballi*.
